# Value Ranges and Clinical Comparisons of Serum DHEA-S, IL-6, and TNF-α in Western Lowland Gorillas

**DOI:** 10.3390/ani12192705

**Published:** 2022-10-08

**Authors:** Ashley N. Edes, Dawn Zimmerman, Balbine Jourdan, Janine L. Brown, Katie L. Edwards

**Affiliations:** 1 Department of Reproductive and Behavioral Sciences, Saint Louis Zoo, St. Louis, MO 63110, USA; 2 Center for Species Survival, Smithsonian Conservation Biology Institute, Front Royal, VA 22630, USA; 3 Veterinary Initiative for Endangered Wildlife, Bozeman, MT 59715, USA; 4 Smithsonian Global Health Program, National Zoological Park, Smithsonian Institution, Washington, DC 20008, USA; 5 Veterinary Teaching Hospital, University of Illinois College of Veterinary Medicine, Urbana, IL 61802, USA; 6 North of England Zoological Society, Chester Zoo, Caughall Road, Upton-by-Chester CH2 1LH, UK

**Keywords:** HPA axis, stress response, immune response, cytokines, reference intervals, zoo, health

## Abstract

**Simple Summary:**

Biomarkers are molecules found in the body that can indicate current physiological functioning and are frequently used to monitor health and diagnose disease. These biomarkers, such as hormones and immune markers, can provide valuable information on the health and welfare of animals. Knowledge on the normal levels of these biomarkers in various species is a crucial step for monitoring health and understanding disease. In this paper, we report assays and value ranges of biomarkers rarely measured in western lowland gorillas in human care. We also compare concentrations of each biomarker between clinical and non-clinical samples. The levels of the two immune biomarkers were higher in clinical samples, but the levels of the neuroendocrine biomarker were not significantly different between clinical and non-clinical samples. These data contribute toward eventually establishing reference ranges for these biomarkers and help improve our understanding of health and welfare in zoo-housed animals.

**Abstract:**

Physiological data can provide valuable information about the health and welfare of animals. Unfortunately, few validated assays and a lack of information on species-typical levels of circulating biomarkers for wildlife make the measurement, interpretation, and practical application of such data difficult. We validated commercially available kits and calculated reference intervals (herein called “value ranges”) for dehydroepiandrosterone-sulfate (DHEA-S), interleukin-6 (IL-6), and tumor necrosis factor-alpha (TNF-α) in a sample of zoo-housed western lowland gorillas due to the roles these biomarkers play in stress and immune responses. For each biomarker, we present species-specific value ranges for a sample of gorillas in human care (*n* = 57). DHEA-S did not vary significantly by sex or age, while IL-6 was higher in males and older gorillas and TNF-α was higher in females but not associated with age. We also compared non-clinical with clinical samples (*n* = 21) to explore whether these biomarkers reflect changes in health status. There was no significant difference between clinical and non-clinical samples for DHEA-S, but both IL-6 and TNF-α were significantly higher in gorillas showing clinical symptoms or prior to death. Additional work is needed to improve our understanding of normal versus clinical variation in these biomarkers, and we encourage continued efforts to identify and validate additional biomarkers that can be used to inform assessments of health and welfare in wildlife.

## 1. Introduction

Reference intervals for physiological biomarkers are important diagnostic and decision-making tools in both human and veterinary medicine [1,2,3,4,5]. Additionally, understanding normal versus atypical levels and fluctuations of biomarkers involved in stress responses can aid in monitoring animal welfare [6,7,8]. One potential biomarker for investigating health- and welfare-related questions in animals is dehydroepiandrosterone (DHEA) and its sulfated form (DHEA-S), which are steroid hormones that provide an index of hypothalamic–pituitary–adrenal (HPA) axis activity [9,10,11,12]. DHEA and DHEA-S (hereafter referred to together as DHEA/S) act as glucocorticoid antagonists, protecting against the deleterious effects of glucocorticoid exposure and, as such, low levels when experiencing stressors are considered maladaptive [9,10,11,12]. DHEA/S also regulate immune function, inhibiting the production of proinflammatory cytokines while increasing the production of anti-inflammatory cytokines [10,11]. In humans, low DHEA-S has been associated with cardiac disease and all-cause mortality [13]. Cardiac disease is the leading cause of death for zoo-housed great apes [14,15]. Given its role in moderating stress and immune responses, interest in measuring DHEA/S in animals has increased in recent years. For example, given the limitations when measuring and interpreting glucocorticoids [16,17,18], Whitham and colleagues [11] recently recommended DHEA/S and their ratio with cortisol be explored as potential physiological indicators of welfare in zoo animals. Previously, we included DHEA-S in allostatic load indices developed for western lowland gorillas [19,20]. Other researchers have shown higher DHEA-S in semi-captive compared to wild orangutans [21] and in Japanese macaque females housed indoors compared to outdoors [22]. In orangutans, DHEA but not DHEA-S was associated with beneficial outcomes in innate immunity [23], but in pigs, there was no difference in DHEA based on parasite infection [24].

Cytokines are proteins that mediate the immune response, providing important information about an individual’s health, and as such, also may help address health and welfare concerns. Proinflammatory cytokines are secreted at the beginning of immune responses, while anti-inflammatory cytokines are released to downregulate the immune response [25]. Proinflammatory cytokines are produced primarily by macrophages, monocytes, and Th lymphocytes [25,26]. Th1 cytokines, such as interleukin-2 (IL-2) and tumor necrosis factor α (TNF-α), regulate cell-mediated immunity against intracellular pathogens (e.g., viruses), while Th2 cytokines, which includes IL-6 and IL-10, mediate humoral immune responses against extracellular pathogens (e.g., parasites) [25]. Due to their short half-lives in the bloodstream, proinflammatory cytokines can be difficult to measure outside of active infections [27,28]. Two commonly measured cytokines, IL-6 and TNF-α, have been associated with insulin resistance, diabetes, and obesity [29,30,31,32], cardiac disease [33,34,35,36], and cancer [37,38,39,40] in humans. These two cytokines also have been associated with stress responses in humans, with increases in IL-6 observed during both acute [37,41,42] and chronic stress [37,38,43], and decreases in TNF-α during chronic stress [44]. IL-6 and TNF-α have been measured infrequently in studies on wildlife. As with DHEA-S, we previously included these two cytokines in allostatic load indices for gorillas [19,20]. We also investigated whether IL-6 and TNF-α predict all-cause morbidity, cardiac disease, and all-cause mortality in gorillas and found that cardiac disease was best predicted by lower TNF-α alongside age and sex [45]. Research in non-primate taxa has measured cytokines in harbor porpoises [46], harbor seal pups [47], Asian and African elephants [28,48], cheetahs [49], and bottlenose dolphins [26]. 

The purpose of this study was to validate commercially available reagents and to calculate value ranges for DHEA-S, IL-6, and TNF-α in a sample of zoo-housed western lowland gorillas. We chose to assay DHEA-S rather than DHEA because it is likely more abundant (i.e., in humans, 99% of DHEA is in the sulfated form) due to its long biological half-life and slow rate of clearance [10,11]. Additionally, unlike DHEA, DHEA-S does not show significant diurnal variation in humans [10,11], chimpanzees [50], or orangutans [21]. DHEA-S, IL-6, and TNF-α are of interest due to the roles they play in stress and immune responses. We calculated reference intervals using standard methodology [4] but refer to them as species-specific value ranges, following our previous work in African and Asian elephants [48], as we are unable to rule out potential underlying health issues without overt clinical signs. In addition to calculating value ranges, we also present a subset of biomarker values for individuals when apparently healthy compared to when they exhibited clinical signs of illness or prior to death.

## 2. Materials and Methods

### 2.1. Subjects, Sample Collection, and Assessment of Health Status

Single serum samples banked during routine veterinary examinations were obtained from 57 (30 male, 27 female) western lowland gorillas, aged 6–51 years $\underset{x}{¯}$ = 21.4, SD = 11.6), housed at four zoos in North America. For 15 gorillas, there were 1–3 additional serum samples collected during veterinary examinations due to the presentation of clinical signs or on the day of death, allowing for 21 comparisons between clinical and non-clinical samples from the same individual. For example, a gorilla with a sample collected during a routine veterinary examination in January 2010 could also have a sample from a root canal in May 2014 and a sample from a laceration in November 2015. That gorilla would then have two clinical comparisons matched with its one routine sample. Following collection, serum samples were stored at −80 °C until transported on dry ice to the laboratories for analysis. Samples were collected between 1992 and 2015. Although sample degradation is a concern with long-term storage, DHEA-S [51,52] and both inflammatory cytokines [53] have been shown to maintain their integrity when cryopreserved without multiple freeze–thaw cycles. This research was approved by The Ohio State University (IACUC #2013A00000147) and the Smithsonian National Zoo as well as each participating institution.

### 2.2. Enzyme Immunoassays

All enzyme immunoassays (EIAs) were conducted using commercially available kits. This project brings together data from two projects focused on investigating allostatic load in zoo-housed gorillas, with the second study based on the findings of the first, and as such, the assays were completed in different laboratories. The assays for the three zoos from the first project were completed by The Ohio State University Center for Clinical and Translation Science: Clinical Research Center in 2014, while assays for the fourth zoo were completed at the Smithsonian Conservation Biology Institute in 2019. When possible, the same commercial kits were used between labs. When there was sufficient volume, samples originally assayed in 2014 were reanalyzed in 2019 with samples from the fourth zoo to compare the datasets from both locations (number of samples compared: DHEA-S, *n* = 33; IL-6, *n* = 49; TNF-α, *n* = 46). We then performed linear regression on biomarker values from each laboratory to ensure they were comparable before combining data (DHEA-S: R^2^ = 0.922; IL-6: R^2^ = 0.740; TNF-α: R^2^ = 0.997; *p* for all <0.001). Values from the fourth zoo were contained within the range of values obtained from the first three zoos for each biomarker. All comparisons between clinical and control samples were from the same institution and thus assayed by the same laboratory using the same assay, ensuring that any differences observed are not due to the normal variation between laboratories. All assays were performed according to manufacturer’s instructions and biochemically validated prior to the start of the study by performing spike and recovery and linearity assessments with gorilla serum. All samples were analyzed in duplicate with coefficients of variation (CV) maintained below 10%; inter-assay CVs were maintained below 15% for high and low concentration controls.

DHEA-S from the first three zoos was assayed using a solid-phase, competitive chemiluminescent EIA (LKDS1, Siemens Healthcare Diagnostics Inc., Hoffman Estates, IL, USA) on an Immulite 1000 Immunoassay System. The calibration range for this DHEA-S EIA was 15–1000 µg/dL. Due to differences in equipment available, samples from the fourth zoo were assayed for DHEA-S using a solid-phase, competitive colorimetric EIA (K054, Arbor Assays, Ann Arbor, MI, USA). The range for this assay was 9.6–6000 µg/dL. Samples were analyzed for DHEA-S at a 1:100 dilution. The immunoassay was validated biochemically for measuring DHEA-S in western lowland gorilla serum through parallelism and matrix interference assessment, and subsequent regression analyses. Serial 5-fold dilutions of serum yielded a displacement curve parallel to the standard curve (y = 1.088x − 6.520, R^2^ = 0.984, F_1,3_ = 184.242, *p* < 0.001). There was no evidence of matrix interference, as addition of appropriately diluted serum (1:100) to assay standards did not alter the amount observed (y = 0.748x + 110.722, R^2^ = 0.998, F_1,3_ = 1772.389, *p* < 0.001).

IL-6 and TNF-α were measured using solid-phase EIAs from the same manufacturer for both sets of samples (R and D Systems, Minneapolis, MN, USA). In each case, the kit used to analyze samples from the first three zoos (IL-6: HS600B; TNF-α: HSTA00D) was discontinued and replaced with an updated kit with increased sensitivity (IL-6: HS600C; TNF-α: HSTA00E) that was used to measure samples from the fourth zoo. The assay range for both IL-6 kits is 0.20–10.0 pg/mL, with sensitivity of the discontinued kit being 0.11 pg/mL and the updated kit being 0.09 pg/mL. Samples were analyzed for IL-6 at 1:5; any that exceeded the highest standard were further diluted (up to 1:40) until they were within range of the standard curve. The IL-6 assay was validated via linearity (82.9%) and spike and recovery (110.3%) assessment within the range of dilutions used. For TNF-α, the assay range on the discontinued kit was 0.5–32.0 pg/mL with a sensitivity of 0.106 pg/mL and on the updated kit is 0.2–10.0 pg/mL with a sensitivity of 0.049 pg/mL. All samples were analyzed undiluted for TNF-α. The TNF-α assay was validated via spike and recovery assessment (124%).

### 2.3. Quantitative Analyses

Species-specific value ranges were calculated for each serum biomarker according to the reference interval guidelines from the American Society for Veterinary Clinical Pathology [4] using the “referenceIntervals” package [54] in R [55], version 4.2.0. Value ranges for all three biomarkers were determined using the robust method, with outliers identified and removed using Cook’s distance. Value ranges are presented for the full dataset as well as separately for males and females; ranges represent 95% of the population and are reported with 90% confidence intervals. 

Potential associations of each biomarker with sex and age in the routine dataset were analyzed using generalized linear models (GLMs) with a gamma distribution and log-link function due to the positively skewed biomarker distributions. A generalized linear mixed model (GLMM) with a gamma distribution, log-link function, and individual entered as a random effect was used to compare each biomarker between samples collected when individuals exhibited active clinical symptoms or on the day of death with a control sample taken from the same individual when no clinical signs were present. Due to the small sample size, differences between those with clinical signs and prior to death were combined for analysis (collectively referred to as “clinical” samples). The GLMs and GLMMs were also conducted in R [55], version 4.2.0, using the “lme4” package [56] with α = 0.05.

## 3. Results

**Figure 1:**
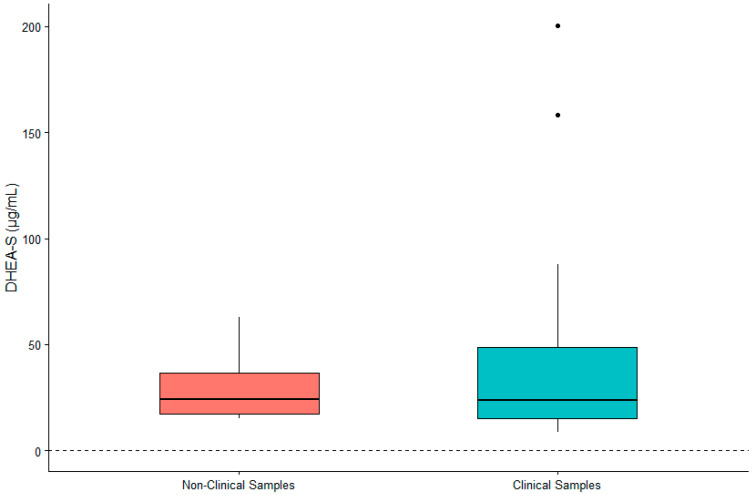
Differences in concentrations of serum DHEA-S between non-clinical and clinical western lowland gorilla (*n* = 21) samples (β = −0.174, SE = 0.109, *p* = 0.110). The calculated lower limit of the species-specific value range is denoted by the dashed horizontal line.

**Figure 2:**
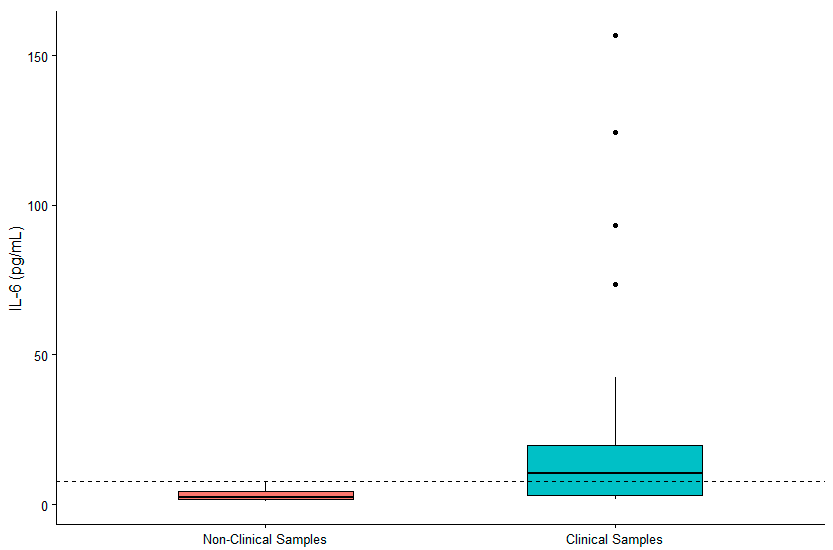
Differences in concentrations of serum IL-6 between non-clinical and clinical western lowland gorilla (*n* = 21) samples (β = −1.661, SE = 0.254, *p* < 0.0001). The calculated upper limit of the species-specific value range is denoted by the dashed horizontal line.

**Figure 3:**
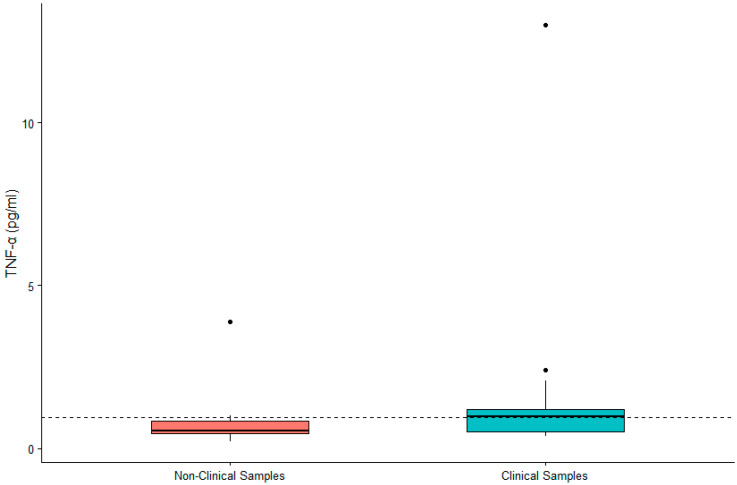
Differences in concentrations of serum TNF-α between non-clinical and clinical western lowland gorilla (*n* = 21) samples (β = −0.461, SE = 0.175, *p* = 0.008). The calculated upper limit of the species-specific value range is denoted by the dashed horizontal line.

DHEA-S was not significantly associated with sex (β = 0.119, SE = 0.167, *p* = 0.480) or age (β = 0.002, SE = 0.007, *p* = 0.790). IL-6 was significantly higher in males (β = 0.442, SE = 0.200, *p* = 0.032) and in older gorillas (β = 0.019, SE = 0.009, *p* = 0.034). TNF-α was significantly higher in female gorillas (β = −0.569, SE = 0.244, *p* = 0.023) but did not vary significantly with age (β = −0.009, SE = 0.011, *p* = 0.392). Value ranges for DHEA-S, IL-6, and TNF-α are presented in Table 1. Given the significant difference between males and females for two of three biomarkers, we present value ranges for the entire sample overall as well as separately by sex. A summary of DHEA-S and cytokine concentrations in individuals (*n* = 21) exhibiting clinical symptoms or prior to death are presented in Table 2. There was no significant difference between DHEA-S in clinical versus non-clinical samples (β = −0.174, SE = 0.109, *p* = 0.110; Figure 1). IL-6 was significantly higher in clinical than non-clinical samples (β = −1.661, SE = 0.254, *p* < 0.0001; Figure 2). Similarly, TNF-α was significantly higher in clinical than non-clinical samples (β = −0.461, SE = 0.175, *p* = 0.008; Figure 3).

## 4. Discussion

Physiological data can provide valuable information about an individual’s health and welfare. Unfortunately, few validated assays and a lack of information on species-typical levels of circulating biomarkers for most species make the measurement, interpretation, and practical application of such data difficult. Herein, we presented validations for three serum biomarkers in western lowland gorillas using commercially available kits. For each biomarker, we then analyzed associations with sex and age, determined species-specific value ranges for western lowland gorillas in human care and compared non-clinical with clinical samples to explore whether these biomarkers reflect changes in health status. For DHEA-S, there were no significant associations with sex or age, as observed previously in a different subset of these data [57]. IL-6 showed a positive association with age and was higher in males, with previous research on a different subset showing a similar age result but no sex differences [46]. The TNF-α results also were consistent with previous research on a different subset of these data, with higher levels in females but no associations with age [46]. When all clinical cases were combined, there was no significant difference between clinical and non-clinical samples for DHEA-S, but both IL-6 and TNF-α were significantly higher in gorillas showing clinical symptoms or prior to death, indicating immune-system activation.

There are no previously published reference intervals for DHEA-S, IL-6, or TNF-α in non-human great apes. In a study investigating DHEA-S in western lowland gorillas in relation to age-related changes and the evolution of adrenarche, average DHEA-S was reported at 22.76 µg/dL [58]. Similarly, in a previous study using a subset of data from this paper, average DHEA-S was 35.5 µg/dL [57]. Both means fall within the middle of the value range calculated herein. Although clinical samples showed more variation around the mean and five values from the clinical dataset were above the upper limit of the value range, differences between clinical and non-clinical samples overall were not significant. In humans, low DHEA-S correlates with increased morbidity and mortality risk [15] and decreased DHEA/S is associated with chronic inflammatory diseases [59]. Conversely, DHEA-S in humans also plays an anti-inflammatory role, including being inversely correlated with IL-6 [60] and inhibiting the production of TNF-α [61,62]. As well as potential associations with immune function, DHEA-S also plays a role in the stress response and is typically upregulated alongside cortisol during stressors [10], for example in orangutans following a stressful event [21]. However, during stressors of an immune nature, cortisol increases independently of DHEA/S as a shift away from androgen synthesis occurs in favor of glucocorticoid production [59]. This complex relationship may explain why, in some cases DHEA-S, was elevated alongside clinical cases of illness or injury, but in the majority, concentrations were within the species-typical value ranges. Alternatively, exposure to chronic stress reduces DHEA-S [11,12], which may result in circulating levels indistinguishable from non-clinical individuals. 

The only previously reported data on inflammatory cytokines in western lowland gorillas is from work we published on allostatic load [19,20] and investigating whether IL-6 and TNF-α predicted morbidity and mortality risk using data from the first three zoos included here [45]. In the aforementioned study, IL-6 did not predict all-cause morbidity, cardiac disease, or mortality risk, but lower TNF-α was associated with increased risk of cardiac disease [45]. In contrast, the present data revealed greater variation around the mean and significant elevations in both biomarkers in the clinical compared to non-clinical samples, although this study focused on clinical events or health declines around the time of death rather than conditions such as cardiac disease. However, these results are consistent with research showing IL-6 and TNF-α are associated with numerous morbid conditions, such as insulin resistance, cardiac disease, cancer, and others in humans [29,30,31,32,33,34,35,36,37,38,39,40,63,64], and acute abdominal disease (colic) in horses [65]. In African and Asian elephants, TNF-α neared being significantly higher in clinical compared to non-clinical cases, but IL-6 did not differ [4]. In Harbor porpoises, neither IL-6 nor TNF-α were associated with severely diseased individuals, those with splenic depletion, or the degree of thymic atrophy [10,12]. 

Studying physiological data obtained from zoo collections, especially across multiple institutions, presents several limitations such as the inability to control factors like diet and husbandry or determine how frequently invasive samples are collected. While we calculated value ranges using the established methodology for calculating reference intervals [23,50,56], the value ranges we present cannot be considered healthy reference intervals due to individuals with incipient or quiet conditions or those with disease medically controlled at the time of sample collection, such as gorillas on cardiac medications. Although we removed statistical outliers, which is a conservative approach recommended when health conditions cannot always be readily diagnosed [38,64], the value ranges we present are likely broader than what would be observed in a truly healthy sample. However, given that it can be difficult to diagnose clinical conditions in zoo-housed species as most wildlife hide clinical signs, the value ranges are likely representative of collections in human care. For each biomarker, the value ranges calculated herein used values from two different laboratories and two different assays or updated kits with higher sensitivities, and variation can occur between labs. However, value ranges are commonly reported using values obtained from multiple laboratories using varying reagents, and when possible, we re-tested samples on the alternative assay to ensure correlation between both datasets. Additionally, as reference intervals are not reliable with fewer than 20 individuals, we were unable to calculate value ranges by age categories. DHEA-S is known to change with age in humans [65,66] and other great apes [20,50,56], and IL-6 is known to increase with age in humans [38,66], suggesting age-based analyses with larger sample sizes may be beneficial to further our understanding of the practical use of these biomarkers. In addition to changes in biomarkers with age, fluctuations in response to variables such as season, diet, social structure, and other factors may present additional confounds for future consideration. Finally, these samples were collected during immobilizations and so biomarker values may be impacted by the anesthetic agents used. For example, ketamine, a commonly used anesthetic agent, has been shown to suppress the release of IL-6 and TNF-α in other species [67,68]. Serum samples collected from individuals trained to voluntarily participate in sample collection would overcome this limitation.

## 5. Conclusions

Serum samples are routinely collected during veterinary examinations and zoos frequently use hormone analysis (performed either in-house or by a service lab) to evaluate their animals, such as measuring glucocorticoids to assess stress responses or monitoring reproductive status with progesterone or testosterone. However, the number of biomarkers available for better evaluation of wildlife species needs to be expanded to help address welfare questions and health concerns. This research is especially critical for identifying sub-clinical conditions, as many species have evolved to hide clinical signs. Here, we report validation, value ranges, and clinical comparisons for DHEA-S, IL-6, and TNF-α in gorillas in human care. These biomarkers may be sensitive to stress- and/or health-related changes that could guide husbandry and veterinary care. Although further work is needed to improve our understanding of variation based on age, sex, and environment (i.e., free-ranging versus captive), this research provides a foundation for future work incorporating these biomarkers. Physiological analyses have the potential to provide powerful data for monitoring the psychological and physical wellbeing of animals in human care, and we encourage continued work to identify and validate biomarkers that can be used to measure welfare and health status across wildlife taxa.

## Figures and Tables

**Table 1:** Descriptive statistics and calculated value ranges (with 95% confidence intervals, CI) for serum biomarkers from 57 western lowland gorillas in human care.

Biomarker	Mean	SD	Median	Minimum	Maximum	N ^a^	Value Range	Lower CI ^b^	Upper CI
DHEA-S (µg/dL)	31.23	18.70	28.60	15.00	123.50	55	0.13–54.84	0.01–8.49	49.38–61.58
IL-6 (pg/mL)	3.73	3.12	2.44	0.49	15.20	55	0.04–7.65		6.27–8.96
TNF-α (pg/mL)	0.59	0.68	0.44	0.11	3.90	55	0.05–0.95		0.80–1.05
Males only (*n* = 30)									
DHEA-S (µg/dL)	32.91	14.75	32.65	15.00	69.00	28	4.43–55.35	0.01–10.19	49.12–62.45
IL-6 (pg/mL)	4.46	3.60	2.89	1.03	15.20	28	0.04–8.82		7.31–10.97
TNF-α (pg/mL)	0.44	0.28	0.38	0.11	1.01	27	0.05–0.81		0.67–0.95
Females only (*n* = 27)									
DHEA-S (µg/dL)	29.37	22.44	21.20	15.00	123.50	26	0.01–49.04	0.01–0.07	39.23–59.06
IL-6 (pg/mL)	2.92	2.29	1.99	0.49	9.72	25	0.04–5.42		4.06–6.82
TNF-α (pg/mL)	0.77	0.92	0.47	0.11	3.90	25	0.05–0.95		0.73–1.08

^a^ Number of samples used for value range calculation after outlier removal. ^b^ For IL-6 and TNF-α, the lower end of the calculated value range is the limit of detection so no lower CI could be calculated.

**Table 2:** DHEA-S, IL-6, and TNF-α concentrations in western lowland gorillas with active clinical symptoms or prior to death. Numbers in bold exceed the upper end of the calculated value range based on all gorillas combined.

Description	Age	Sex	# of Months from Routine Sample	DHEA-S (µg/dL)	IL-6 (pg/mL)	TNF-α (pg/mL)
Active clinical symptoms						
Normal parturition, healthy baby, maternal neglect	14	F	37	**87.70**	**19.78**	**2.08**
Root canal	15	F	19	44.33	4.59	0.91
Root canal, finger laceration; known mild cardiac disease	22	M	32	35.81	**10.44**	**0.98**
Root canal	24	F	30	26.51	5.47	0.66
Significant dental disease; multiple dental extractions	28	F	82	26.22	2.71	0.80
Constipation, lethargic, decreased appetite 3 month duration	23	M	8 *	19.20	1.86	**1.00**
Preshipment and biannual exam, chronic loose stools; history of intestinal resection for Balantidium enteritis	11	F	98 *	15.00	**10.43**	0.40
Colonoscopy for diarrhea; known cardiac disease	27	M	10	15.00	1.63	0.37
Distended abdomen, mild exercise intolerance, diagnosed with right-sided congestive heart failure	43	F	68	**82.71**	3.04	**1.19**
Weight gain despite dietary reduction, slightly distended abdomen; known cardiomyopathy and hypothyroid	26	M	15 *	**60.60**	**19.69**	0.38
Recheck bloodwork (leukopenia); known cardiac disease	30	M	47	15.00	1.63	0.37
Acute onset stiffness, mild exercise intolerance	40	F	39	**200.43**	**124.22**	**13.00**
Right leg lameness (significant coxofemoral arthritis)	39	F	27 *	15.5	5.63	0.53
Non-healing abscess on shoulder; known hypothyroid	19	F	33	15.00	2.03	0.39
Deep laceration to left forearm; known hypothyroid	20	F	48	15.00	**11.10**	0.58
Right forelimb/hindlimb lameness, fractured right clavicle and radius; known hip arthritis and hypothyroid	48	F	14	15.00	**10.89**	**1.16**
Chronic weight loss, intermittent cough non-responsive to antibiotics, diagnosed with congestive heart failure; 10 days prior to death	23	M	35	23.48	**156.88**	**1.80**
Samples collected day of death						
Immobilized for cardiac resynchronization therapy; died	23	M	35	8.75	**73.66**	**1.77**
Coughing for one month, chronic stiffness; known cardiac disease; acutely collapsed during social introduction	34	M	36	48.70	**42.32**	**1.13**
Euthanized, diagnosed with right-sided congestive heart failure December 2010	43	F	71	**158.21**	**93.32**	**2.41**
Immobilized for lethargy and increased respirations; did not recover; preliminary necropsy results show mild gastritis, small bowel enteritis, possible pancreatitis, and pleural effusion/pulmonary edema (possibly from CPR)	50	F	28	23.80	**14.2**	**0.98**

Routine samples preceded those collected during active clinical symptoms or prior to death unless marked with an *.

## Data Availability

Data are available from the corresponding and second authors upon reasonable request.
